# Exploring public health nurses’ acceptability of clinical assessment tools in a Norwegian child health centre

**DOI:** 10.1017/S146342362400001X

**Published:** 2024-02-12

**Authors:** Elisabeth Ovanger Barrett, Hilde Laholt, Geir Fagerjord Lorem, Catharina Elisabeth Arfwedson Wang

**Affiliations:** 1 Faculty of Health Sciences – Department of Psychology/Specialist in Clinical Community Psychology, UiT – The Arctic University of Norway, Municipality of Tromsø, Norway; 2 Faculty of Health Sciences – Department of Health and Care Sciences, UiT – The Arctic University of Norway, Municipality of Tromsø, Norway; 3 Faculty of Health Sciences – Department of Psychology, UiT – The Arctic University of Norway, Municipality of Tromsø, Norway

**Keywords:** ADBB, assessment tools, child health centre, focus group discussion, implementation in primary health care, infant follow-up, public health nurses

## Abstract

**Background:**

Infants’ symptoms of mental struggle are often diffuse and undifferentiated, and health services do not identify many infants at risk of poor development. However, primary health care is advantageous for early identification, given there are frequent consultations during the infant’s first two years. Health policy encourages using evidence-based screening but use varies in primary health care. The Alarm Distress Baby Scale (ADBB) is an assessment tool targeting social withdrawal in infants 2-24 months of age.

**Aim:**

To explore contextual factors related to public health nurses’ (PHNs) acceptability of clinical assessment tools in a Norwegian child health centre.

**Methods:**

Prior to an upcoming ADBB training, we used focus group discussions with PHNs to explore their views on their professional role and practice and how this concurs with using assessment tools.

**Findings:**

Thematic analysis resulted in the following themes: (1) A Role requiring Supporting the Parents and Safeguarding the Infant; (2) The Challenge of Interpreting Infant Expressions; and (3) Organisational Preconditions for Accepting New Methods.

**Conclusion:**

Our findings show that PHNs regard assessment tools as an aid to detect infants at risk, but that systematic use of such tools can hinder their ability to be flexible, egalitarian, and resource-focused. We also find that acceptability of assessment tools requires a system for continuous training and a well-established referral routine.

## Introduction

Infancy is a critical time with rapid, formative brain development and a dependence on attentive caregivers for healthy growth. Mental health problems in early childhood are as prevalent as in older age groups, but many infants at risk of poor development are neither identified nor treated (Egger and Angold, 2006; Skovgaard *et al.*, 2007; Bagner *et al.*, 2012; Wichstrøm *et al.*, 2012; Charach *et al.*, 2020). Infants express mental health problems in terms of sustained regulatory disturbance, for example, uneasiness or social withdrawal, which can negatively affect the child’s development and parent-child interaction (Møller-Pedersen, 2010). Accordingly, special consideration from the help services is important to identify infants at risk (Lyons-Ruth *et al.*, 2017), where primary health care plays a crucial role.

### The Norwegian child health centre

The Norwegian public primary healthcare system for children offers regular health check-ups at a child health centre (CHC) (0-5 years) as part of children’s right to health care (‘Act on municipal health and care services’, 2011). The CHC is low-threshold, free-of-charge and used by most families. Its aim is to facilitate physical and mental health, good environmental conditions and prevent harm and illness as early as possible (Helsedirektoratet, 2017). The CHC summons the infant to 13 routine consultations during the first two years and offers regular check-ups with a physician. The national guideline for the CHC recommends collaboration with physiotherapists, psychologists, and other health professionals if needed. These numerous and multidisciplinary examinations make the CHC a unique area for the early identification of infants at risk of poor development, and the guideline emphasises the detection of child abuse and neglect (Helsedirektoratet, 2017).

The PHNs are the leading healthcare providers in the CHC. Public health nursing originates from the 1920s, oriented towards prevention and control of infectious diseases with a top-down authoritarian role as health inspector working in collaboration with the district doctor (Schiøtz, 2003; Clancy, 2007; Dahl *et al.*, 2013). Today, the PHNs’ role has broadened from physical follow-up of the child’s development to supporting the health and well-being of the whole family by emphasising empowerment (Schiøtz, 2003; Clancy, 2007). The CHCs’ responsibility in promoting physical and mental health and preventing abuse and neglect is clearly stated in governmental documents and guidelines, including training health personnel in assessment tools and methods of dialogue (Helsedirektoratet, 2017).

### Early identification of infants at risk

Traditional methods of assessing socio-emotional development problems in primary health care for children are screening, observation, and clinical judgement based on knowledge about the child’s development (Valla and Olavesen, 2016; Huffman and Baran, 2019). Studies show higher detection rates of autism and developmental problems when using evidence-based screening tools (Thomas *et al.*, 2016; Sánchez-García *et al.*, 2019) and increased odds of receiving mental health treatment only when using a combination of monitoring and screening procedures (Barger *et al.*, 2018). Some recommend greater use of screening programmes (Bagner *et al.*, 2012; Glascoe, 2015; Lipkin and Macias, 2020; Sheldrick *et al.*, 2020); however, research shows divergent results. One systematic review on identifying preschool children with mental health problems in primary health care points to sparse literature and variation in methods’ quality (Charach *et al.*, 2020), while another reveals that evidence regarding psychometric properties of psychological tests is often lacking (Breivik *et al.*, 2021). Others point to drift from screening guidelines (Wissow *et al.*, 2013; Gellasch, 2016; Wallis *et al.*, 2020) and low screening rates (Hirai *et al.*, 2018).

Professionals worry that assessment tools can compromise their role in empowering and promoting good parental practice (Ersvik, 2012). Others are reluctant to systematically assess symptoms because of limited service and referral options (Noonan *et al.*, 2017). Gellasch (2016) conducted a review of qualitative studies of parents’ and providers’ (mainly paediatricians’) experiences of developmental screening in primary health care. Providers pointed out barriers, including lack of time and reimbursement, fear of creating unnecessary worry, and lack of screening education. Parents described a lack of time to complete instruments and challenges in the parent-provider partnership, such as lack of responsiveness or false reassurance from the providers, when raising their concerns. The review further highlighted the underrepresentation of advanced practice nurses concerned with developmental screening.

### The Alarm Distress Baby Scale

The Alarm Distress Baby Scale (ADBB) (Guedeney and Fermanian, 2001) assesses social withdrawal in infants 2–24 months, designed to fit a CHC setting where the scale is integrated in the clinical examination. Sustained social withdrawal is a regulation difficulty and a critical alarm signal that can have both organic causes and be related to caregiver relationship disturbances (Guedeney and Fermanian, 2001). The prevalence of sustained social withdrawal is around 3 % in a non-clinical population (Puura *et al.*, 2010), but higher in clinical-referred infants (Dollberg *et al.*, 2006).

Smith-Nielsen *et al.* (2018) studied the feasibility of implementing ADBB in primary health care in Denmark. They found that 79% of the children had at least one ADBB screening 12 months post-implementation and that health visitors’ attitude towards the ADBB significantly predicted screening prevalence rates. When directing future research, they suggested qualitative interviews to gain in-depth insight into context-related factors related to the implementation process.

This pre-implementation study aimed to supplement previous implementation research on ADBB, enhancing our knowledge of contextual factors related to the PHN’s acceptability of assessment tools in primary health care for children. We define contextual factors as aspects of the PHN’s role and practice, such as their target group, tasks, and responsibility. Specifically, we wanted to gain in-depth understanding of (1) how the PHNs perceive their role and practice with infants in the CHC, (2) how this concurs with using systematic assessment tools, and (3) expectations regarding the upcoming ADBB training.

## Method

This was a qualitative phenomenological study with a descriptive design, using focus group discussions (FGD) to explore the range of experiences, opinions, and perceptions that the participants had about utilising screening instruments in general and ADBB in particular (Krueger and Casey, 2015). A phenomenological approach allowed for the enrichment of data as a result of participants sharing and discussing experiences and enabled clarification between participants and the participant and researcher (Bradbury-Jones *et al.*, 2009).

### Setting and recruitment of participants

The municipality of Tromsø has five CHCs located in different districts. We utilised purposeful sampling by asking all the PHNs working with infants and embarking on ADBB training and certification to participate. We met with the lead PHNs at the CHCs, informing them about the study’s aim and the request for participation. The lead PHNs informed the other PHNs and handed out written information and informed consent forms. Signed consent forms were returned to the lead PHN. None of the PHNs declined participation. The majority of the PHNs had many years of experience with infant follow-up and training in assessment tools, including the Edinburgh Postnatal Depression Scale (EPDS) (Cox *et al.*, 1987) and Ages and Stages Questionnaire (ASQ) (Squires *et al.*, 1999). We scheduled one FGD at each of the five CHCs with 4–6 participants in each group (Table 1). Four of the PHNs were absent on the day of the FGD, giving us a total of 25 participants.

**Table 1.** Overview of attendees in the focus group discussions

Focus group (in date order)	No. of attendees	Experience range in years
1	6	0–19
2	6	3–32
3	4	13–21
4	4	5–14
5	5	1–13

### Data collection

We collected the data in September 2019. The FGDs lasted between 72–91 min. The first author (EOB) moderated the FGDs with supervision from the second author (HL), a PHN and researcher experienced in conducting FGDs. We introduced the FGDs by explaining the study objectives. We had a questioning route (QR) designed to explore our research aim with four topics regarding the PHNs: a) role in infant follow-up, b) experiences with detecting infants at risk of a poor developmental trajectory, c) experiences and attitudes towards assessment tools, and d) expectations regarding the upcoming ADBB training. We encouraged an open discussion and sharing of different perspectives and experiences, and we used the QR flexibly. When asked about how the PHNs experienced participation, the PHNs reported that they found discussing professional issues valuable. The FGDs were audio recorded and transcribed verbatim.

### Data analysis

We analysed the data using Inductive Thematic Analysis (ITA) (Braun and Clarke, 2006). ITA has clear guidelines for performing the analysis and considers researcher subjectivity as a resource and meaning as situated and contextual (Clarke and Braun, 2018). ITA consists of six phases, which we operationalised as follows: We immersed ourselves in the transcripts through repeated reading, checked each transcript against the audio files to ensure accurate transcriptions, and marked ideas for coding. We used NVivo12 (*QSR International Pty Ltd*, 2018) to organise the data and coded the transcripts segment by segment. Regular meetings between the primary coder (EOB) and secondary coder (GL) enabled discussions around analytic approaches and explorations of ideas and interpretations. We examined the codes and grouped those that formed broader patterns of meaning into candidate themes. We checked candidate themes against the transcripts, pursuing a good fit. In the later stages of analysis, we discussed the themes and results with all the authors and presented them to members of the PHNs’ professional field for feedback. The presentation of findings includes illustrative participant quotations. We edited the quotes from spontaneous oral speech to readable written text to facilitate comprehension.

## Findings

The findings are organised into three main themes with associated subthemes (Table 2). The PHNs’ role in the CHC is dual and complex, balancing parental support and safeguarding infants whose expression is difficult to interpret. The PHNs fear that using assessment tools could contradict their role as parent support; on the other hand, it could elucidate infant expression and help the PHNs act when worried instead of waiting and seeing. The results further indicate that the PHNs experience an absence of necessary organisational premises that can compromise the acceptability of assessment tools.

**Table 2.** Main themes with associated subthemes

A Role Requiring Supporting Parents and Safeguarding Infants	The Challenge of Interpreting Infant Expressions	Organisational Preconditions for Accepting New Methods
Baby-centred parental support	Infant-parent dyad	Time and resources to learn and maintain new competencies
Assessment tools can contradict their role as parent support	Infant expressions are ambiguous and vague	A well-established referral routine
Balancing infant advocacy and parent support	Assessment tools can elucidate infant expressions	

### A role that requires supporting parents and safeguarding infants

The PHNs described their role as dual and complex, with responsibilities supporting and empowering the parents and safeguarding the infant. The PHNs feared that using assessment tools could contradict their role as parent support.

#### Baby-centred parental support

The PHNs’ primary concern was the baby’s health, viewing the parents as central to the baby’s health but secondary to the primary purpose of their practice.

‘*The emphasis is on the child’s best interest. Sometimes we see that the parents are not acting in the child’s best interest, and then we must do something – we try different things to help the parents improve to ensure healthy development for the child’*. (FGD4, part. 3)

The PHN discussed how they monitored the infants’ health and development and observed the interplay and attachment between the infant and its parents.

‘*It is like being a link between the infant and its parents’*. (FGD1, part. 5) *and ‘We add knowledge (to the parents) that creates a better understanding (of the infant)’*. (FGD4, part. 3)

The PHNs convey the status of the infant’s development, keeping the parents informed about the competencies and needs of their child. It allows the parents to fine-tune their praxis accordingly and seek further help if needed.

The PHNs described the importance of attending to the parents’ health and well-being, offering support and guidance.

‘*Sometimes we see how the parents themselves are struggling or experiencing much stress. Then we help them see the resources they have that can help them take care of their child, and we help them see that it is ok to ask for help to make ends meet’*. (FGD4, part. 2)

This further highlights how the PHNs support the parents to ensure the infants’ best interest, and they adjust their role to meet the infants’ needs.

‘*Some need much support while others cope so well that we ought not to interfere’*. (FGD1, part. 1) and ‘*We are flexible and offer different kinds of help and support so that the parents, in turn, can choose the kind of help that best suits them’*. (FGD4, part. 1)

The PHNs perceive the parents as inherently capable of parenting, given that they receive the necessary information and support. Maintaining a good relationship with the parents was viewed as crucial.

‘*Our role is to give much support to the parents so that they can take good care of their child, giving the child the best possible odds, and focusing on the positive things’*. (FGD2, part. 6)

#### Assessment tools can contradict their role as parent support

The PHNs were concerned about how using assessment tools could be like ‘ticking boxes’, compromising the relationship with the parents.

‘*When we used the ASQ routinely, a mother commented: well, this is more or less like a MOT-test’*.^1^ (FGD2, part. 1)

They were concerned about how assessment tools could entail a problem-focused rather than resource-focused role. They described the importance of being flexible and tailoring their support to the family’s needs rather than having a pre-set agenda.

‘*What if I know a family well and I know everything is in place? The mother wants to discuss something important to her, but because the routine says I need to administer this assessment tool, we will not have time to discuss her issue’*. (FGD1, part. 5)

#### Balancing infant advocacy and parent support

The PHNs discussed scenarios that would make them worry about the infants’ health and well-being, such as repeatedly missed appointments, parents trivialising PHNs’ concerns, or parents failing to carry out agreed-upon interventions.

‘*Being concerned about the parental care and offering the parents different interventions, but the parents refuse – that can increase my worries. Because then I see them fall short on something, but they do not see it themselves’*. (FGD 4, part. 2)

What characterised PHNs’ worry was not the content of the concern itself but the parents not sharing their concerns, for example, situations where they repeatedly observed problematic behaviour by the parents when parents interacted with the infant, or deviant infant development, such as a flattening growth curve.

In cases like this, the PHNs described having a responsibility as the infant’s ‘*advocate’*.

‘*We have an important role in ensuring that the infants get the upbringing they are entitled to’. – ‘…because they do not have so many ways of letting us know (that they are not ok)’*. (FGD 4, part. 3 and 4)

The PHNs discussed their responsibility to be attentive to signs of maltreatment,

‘*(…) I reckon this job is a huge responsibility. It is rewarding and nice and everything, and most of the time, gladly things are very good. However, in those situations where you are unsure and worried, I think it is heavy duty’*. (FGD 5, part. 5)

The PHNs described moving from an indirect to a direct role in caring for the infant as momentous and challenging, being the only public institution that encounters the infant regularly during its first year of life. The PHNs described a shared experience of uncertainty and ambivalence when deciding whether to take direct action towards safeguarding the infant, risking compromising the parents’ trust.

‘*We report least on the youngest children. All the statistics and our own experience confirm this. Child welfare reports more often concern school-age children than infants because schoolchildren can talk for themselves (sighs), and I have often thought that I should not have waited so long, I should have seen it before, and I should have acted earlier’*. (FGD4, part. 3)

Having to balance the roles of parent support and infant advocacy seems to keep the PHNs often operating in a grey zone.

‘*When we offer support, and the parents hesitate, you feel lost. When you think it could be useful to intervene, but the parents do not think the same, what are you supposed to do then?’* (FGD 3, part. 4)

### The challenge of interpreting infants’ expressions

The PHNs described uncertainty regarding how to interpret infants’ expressions due to the infant-parent dyad and the expressions’ ambiguous and vague nature. The PHNs described how using assessment tools could elucidate infants’ expressions.

#### Infant-parent dyad

The PHNs described how they always encounter infants and parents together. This was viewed partially as valuable because of the inter-relatedness of infant and parent experiences and because it enabled the assessment of attachment and interplay. The PHNs discussed how their attention was divided between PHNs’ and parents’ concerns.

‘*I find that the symbiosis between the infant and the parent can make it difficult to focus on the child on its own – because what you see is this unity. The older they get, the more I think you get a more isolated view of the child…’ – ‘…yes that is how it is, because then you have a completely different communication with the child, whereas now it is via the parents…’ – ‘…yes, a lot has to do with what the parents tell you and what they present in the consultation’*. (FGD 1, part. 5, 3 and 1)

Having the parents always present makes it difficult for the PHNs to gain ‘direct access’ to the infant, with the potential that the infant in its person becomes peripheral when it should be right in the foreground.

#### Infants’ expressions are ambiguous and vague

Experiencing a lack of social contact with the infant created worry in the PHNs.

‘*If you do not make eye contact, I worry. I once encountered an infant I thought was blind, but it turned out that there was nothing wrong with the infant’s vision – the problem was the parent-infant interaction. That really opened my eyes to how things can develop’*. (FGD 5, part. 4)

A shared experience amongst the PHNs was finding infant expressions ambiguous.

‘*I find it challenging understanding the so-called colic kids – knowing whether it is actually colic or whether the infant cries because the parents are exhausted, and they have entered a vicious circle’*. (FGD 5, part. 3)

When discussing being worried about infants, the PHNs often discussed this worry as a ‘*gut feeling’* or ‘*sensing’* that something was wrong.

‘*Well, I think you can have this feeling of something striking a discordant note but not being able to grasp what it is. Nothing concrete or manifest allows me to report to the child welfare service, but you get this feeling of something appearing strange. That is something I have felt’*. (FGD1, part. 2)

The PHNs described infant expressions as difficult to operationalise and write up in patient journals and referral documents. They also described the interpretation of signals and distinguishing normal reactions from alarm signals as complex.

‘*(…) them being so young, and sometimes it is difficult to figure out the situation. Understanding all the signals is difficult, and we know that if a child expresses clear symptoms, things can have been wrong for quite some time’*. (FGD 5, part. 5)

#### Assessment tools elucidate infant expressions

The PHNs hoped that an assessment tool like the ADBB scale could aid them in attending to the infants’ health and well-being and in conveying their observations to the parents.

‘*…it can enhance our competence in understanding the infants and their signals. It can make it easier for us to verbalise and describe our observations’*. (FGD4, part. 4)

Being more concrete and explicit in their observation could make the parents more conscious and knowledgeable about their infant’s developmental needs. For example, one PHN described her experience with the ASQ as follows: ‘*We often got positive feedback from the parents, in that they could really see what their children managed’.* (FGD1, part. 1)

The PHNs discussed how assessment tools could help them verbalise a vague worry and contribute to the PHN feeling confident enough to act instead of to wait and see.

‘*But I think that for our role as an advocate for the infant, using an assessment tool like ADBB is very helpful because then you give clearer feedback regarding your observations. Maybe that makes us act earlier instead of waiting and seeing as we often do. That’s my hope anyway, that we are confident enough to step out of the role of this “family-something” and step up for the infant…’* (FGD1, part. 6)

Using assessment tools could contribute to consensus and a common language between colleagues and facilitate collaboration with other professionals. Assessment tools could help verbalise observations when making referrals to other services and increase their professional confidence.

‘*You might feel more confident when making a referral because you have followed a credible systematic procedure. I think this could be helpful’*. (FGD 3, part. 2)

### Organisational preconditions for accepting new methods

When discussing expectations regarding the upcoming ADBB training, organisational preconditions for accepting new routines were central. The PHNs considered it essential to have the time and resources to learn and maintain the new knowledge and competencies as well as a well-established referral system.

#### Time and resources to learn and maintain new competencies

The PHNs experienced a heavy workload with a packed schedule that compromised professional development,

‘*Well, it is a matter of prioritising because you always fall behind on something. I always have something that needs attending first, and before I know it, the day is gone’*. (FGD2, part. 6)

The PHNs discussed that having leaders allocating time and resources designated to training and supervision was necessary for their motivation to acquire new knowledge and that new routines ought to be helpful and valid.

‘*I hope the ADBB will be viewed as a helpful tool and not just yet another thing we have to do. We have had a lot going on this year, new routines, and more consultations. I think it has been burdensome for us – so I hope this will be seen as something positive that can help us’*. (FGD2, part. 2)

Another critical issue was ensuring that acquired competence was maintained in the institution.

‘*…the drawback regarding all these assessment tools is how we use many resources on a one-time training, not planning for future training sessions for new colleagues. In this way, the competence dilutes, and we might diverge from the supposed usage (…). It makes me feel insecure – am I doing this right? Is this how it is supposed to be?’* (FGD5, part. 5)

#### A well-established referral routine

A common concern regarded managing worrisome scores on assessment tools.

‘*…to be confident in detecting risk, you need to know what to do next’*. (FGD1, part. 5)

The PHNs described having a well-established follow-up system as an essential contextual framework.

‘*How do we manage our worry, how do we talk to the parents about it, how do we follow up, who do we cooperate with – all these things need to be in place’*. (FGD2, part. 2)

The PHNs also discussed how detecting worrisome scores and communicating these to the parents could yield uncertainty and discomfort, as well as the importance of having standard guidelines and a system of guidance, supervision, and referral to feel confident when managing scores on assessment tools.

## Discussion

This pre-implementation study has given insight into the PHNs’ role and practice in the CHC and how clinical assessment tools concur with this setting. It supplements previous implementation research on ADBB and contributes to the knowledge of contextual factors related to the acceptability of assessment tools in primary health care for infants. Our study shows that an important premise for accepting clinical assessment tools like ADBB is that their use attunes with the professionals’ role identity, which in this case regards supporting the parents and safeguarding the infant. Another important premise is that assessment tools contribute to the quality of the PHNs’ work. In our study, using an assessment tool like the ADBB scale is seen as an aid in interpreting infant expression. Finally, acceptability of assessment tools requires that the tools are included in the service’s plans for development and training, as well as an available referral option.

The findings concerning the tricky balancing act between infant advocacy and parent support are in line with a previous review study showing the importance of child health nurses working in partnership with parents (Wightman *et al.*, 2022) and describing the tension between a primary role of supporting families while simultaneously monitoring and policing them (Briggs, 2007; Lines *et al.*, 2017). Our finding that using assessment tools can contradict the PHNs’ role as parent support by hindering flexibility, egality, and resource focus resonates with previous research. Gellasch (2016) described professionals’ fear of creating unnecessary worry when using developmental screening, and Rollans *et al.* (2013) found that child and health nurses experience discordance between the state policy requirement of incorporating structured depression screening procedures and their professional adoption of a more flexible and relationship-based approach to assessment.

Another important finding in our study was the PHNs’ description of challenges in interpreting infant expression. This finding is consistent with research regarding a nurse’s role in keeping children safe (Lines *et al.* 2017) where vague signs and suspicions limited to a ‘gut feeling’ created uncertainty in taking action to ensure the child’s well-being because of concern about drawing wrong conclusions. Fraser *et al.* (2014) have reviewed the literature on child and family health nurses’ role and experience and points to nurses’ concerns about educational preparedness and competence in detecting infant developmental difficulties.

Given the challenges in interpreting infant expression, an assessment tool like the ADBB scale could be essential. Enhanced competence in understanding, concretising, and verbalising infant expression can facilitate infants’ follow-up within the CHC and ensure referrals to more specialised help services when needed. Today, infants 0–2 years rarely receive treatment within specialised psychological and psychiatric services (Bagner *et al.*, 2012; Bremnes and Indregård, 2021), and many infants and children suffering from maltreatment remain undetected (Bufdir, 2015; WHO, 2022).

In our study, time and resources to learn and maintain new competencies were an important precondition for using new methods – findings consistent with other studies (Lau *et al.*, 2016; Gellasch, 2019). A Norwegian status report on public health nurses (Lassemo and Melby, 2020) shows an occupation marked by too few, and often part-time- and project-based, employees with heavy workloads, making it challenging to organise and prioritise professional development within a service. This is grave, given the PHNs’ huge and complex area of responsibility. Enabling a health service to hold both clinical tasks and implement new methods requires having enough staff and resources to carry out training that involves supervision and planning for maintenance. Lau *et al.* (2016) highlight the importance of a good ‘fit’ between the intervention and context in which it takes place. A lack of policy and legislation, incentivisation structures, infrastructure, available resources, and staff involvement are some of the most common barriers.

To summarise, the PHNs’ role is highly complex, balancing parent support and infant advocacy. Additionally, the government has clear expectations regarding the CHC’s role in detecting infants at risk. Altogether, this significantly demands PHNs’ competence. The PHNs need sufficient training that exceeds instrumental administration, including how to use assessment tools in a way that upholds their dual role as an infant advocate and parent supporter. Moreover, enough support, for example, a multidisciplinary workforce available for professional discussions and guidance, and more specialised health services available for referral, are important, both findings consistent with other studies, for example, Gellasch (2016) and Noonan *et al.* (2017).

## Conclusion and implications for practice and policy

The complexity of tasks and responsibilities within the CHC requires further political and educational initiatives to build a full service. Learning and using clinical assessment tools in the CHC can enhance PHNs’ competence in infant health and development and make the PHNs stand more firmly and confident when balancing their dual role. However, acceptability towards assessment tools requires an adequate level of organisational support and referral options.

## Limitations and directions for further research

In this study, all the participants were from the same geographical area and health service, which might limit the generalisation of findings to other child healthcare settings. However, the findings resonate with international literature on the implementation of evidence-based methods in primary health care. An important scope for further research is exploring management and client perspectives to gain a more comprehensive view on this topic.

## Note

1. A MOT test is a Ministry of Transport test, an annual test of vehicle safety and roadworthiness.
